# Prevalence, Pathogenesis, Antibiotic Susceptibility Profiles, and In-vitro Activity of Selected Medicinal Plants Against *Aeromonas* Isolates from Stool Samples of Patients in the Venda Region of South Africa

**Published:** 2007-12

**Authors:** C.L. Obi, J. Ramalivhana, A. Samie, E.O. Igumbor

**Affiliations:** 1 College of Agriculture and Environmental Sciences, School of Agriculture and Life Sciences, University of South Africa, PO Box 392, Sunnyside, Pretoria, UNISA 0003, South Africa; 2 Department of Microbiology, University of Venda, Private Bag X5050, Thohoyandou, Limpopo Province, South Africa

**Keywords:** *Aeromonas*, *Aeromonas hydrophila*, Antibiotic resistance, Antibiotics, Microbial sensitivity tests, Plants, Medicinal, South Africa

## Abstract

The prevalence, pathogenic indices, such as haemolytic and haemagglutinating activities, antibiograms, and in-vitro activities of local medicinal plants against *Aeromonas* isolates in Vhembe district of Limpopo province, South Africa, were studied using standard microbiological methods. In total, 309 diarrhoeic stool samples were collected from patients attending five health centres in the region during December 2004–May 2005. *Aeromonas* species were identified using the API 20E system. The haemagglutinating and haemolytic activities of isolates on human, sheep, pig and chicken red blood cells were investigated. Antibiotic susceptibility profiles of the isolates to several antibiotics and in-vitro activity of local medicinal plants were also ascertained using previously-reported schemes. Results showed that 104 (33.6%) of the 309 samples were positive for *Aeromonas* species, of which 89 (85.6%) were *Aeromonas hydrophila*, 12 (11.5%) *A. sobria*, and three (2.9%) *A. caviae*. All strains of *A. hydrophila* and *A. caviae* produced haemolysis on sheep blood, while eight of the 12 *A. sobria* strains were haemolytic on sheep blood. The haemolytic activities of the isolates were variable on other red blood cells tested. High level of resistance was observed to amoxicillin and ampicillin, followed by cefuroxime (79%), chloramphenicol (74%), and erythromycin (65%). The carbapenems were the most active drugs with only 7% resistance to meropenem and 11% to imipenem. About 12% of the isolates were resistant to ciprofloxacin. The extracts of three of seven medicinal plants tested showed inhibitory activity against all *Aeromonas* isolates; these included acetone and hexane extracts of *Pterocarpus angolensis, Syzygium cordatum*, and *Zornia milneana*. The results suggest a high prevalence of *Aeromonas* species in the region. The isolates demonstrated multiple resistant profiles to different antibiotics tested. Some local medicinal plants were inhibitory to *Aeromonas* isolates, indicating a potential role in the management of *Aeromonas*-related infections. Structural elucidation of the active components may pave the way for the discovery of candidate templates for eventual drug design. Most isolates possessed important virulence characteristics based on their haemolytic and haemagglutinating ability. However, the genetic characterization of the isolates will further confirm their pathogenicity and the origin of multiple antibiotic resistance.

## INTRODUCTION

*Aeromonas* spp. are pathogenic in fish and several cold-blooded animals (1,2). Over the last few years, interest in *Aeromonas* spp. has gone beyond the boundaries of fish pathology due to the surge of diseases in humans caused by it. Seven *Aeromonas* spp., currently recognized as human pathogens, include *Aeromonas hydrophila, A. caviae, A. veronii* biovar *sobria, A. veronii* biovar *veronii, A. jandaei, A. trota*, and *A. schubertii*. Other species, such as *A. eucrenophila*, were incriminated as a cause of fluid accumulation in the rabbit ileal loop test after serial passages, indicating a pathogenic potential (3). It has also been demonstrated that at least one strain of *A. trota* produces aerolysin (4). Although *Aeromonas* species are opportunistic pathogens for humans, studies have shown that they may also act as primary pathogens for humans in a number of infections (4).

*Aeromonas* spp. are distributed worldwide and have been implicated in various infections, including diarrhoea and extraintestinal infections, such as septicaemia, wound infections, burn-associated sepsis, and respiratory tract infections (5–7). Aeromonads were implicated in cases of gastrointestinal infections in children and in wound infections (8–12). Although incriminated in several disease states, the mechanisms of pathogenicity of aeromonads are not clearly understood, and several virulence factors have been proposed. These include the production of endotoxins, extracellular enterotoxins, haemolysins, haemagglutinins, cytotoxins, proteases, and siderosphores and the ability to adhere to cells and possession of certain surface proteins (13,14). Studies have revealed the close association between the expression of a cell-free haemolysin by aeromonads and enterotoxigenic activity (13–15). Various studies also indicated that enterotoxigenic strains of *Aeromonas* strongly produce haemagglutinins (16,17). Therefore, haemolysis and haemagglutinating activities could be strong indicators of enterotoxigenicity by *Aeromonas* species in humans.

Although infections due to *Aeromonas* may be selflimiting, treatment with antibiotics is generally necessary to curb the progression and persistence of the disease, particularly in vulnerable groups, such as the young, elderly, and immunocompromised individuals. The growing antibiotic resistance of pathogenic bacteria worldwide is a compounding factor for the effective management of bacterial infections. An increase in antibiotic resistance of the genus *Aeromonas*, particularly to antibiotics, has been reported (1,17–19). Attempts have been made to elucidate the occurrence and persistence of antibiotic resistance among isolates but this was mainly on aquatic species (20,21). Relatively, low attention has been accorded to the study of antibiotic resistance among human isolates of Aeromonads, particularly in Africa. The need for the periodic monitoring of occurrence of *Aeromonas*-associated infections and their susceptibility to commonly-used antibiotics is urgent for effective healthcare delivery. The present study evaluated the prevalence, pathogenic indices, antibiotic susceptibility profiles, and in-vitro activity of medicinal plants against *Aeromonas* isolates from diarrhoeic stool samples of patients attending five major health centres in Vhembe district, Limpopo province, South Africa.

## MATERIALS AND METHODS

### Study site and patients

The study was carried out in Vhembe district in the Venda region, Limpopo province of South Africa during December 2004–May 2005. Diarrhoeic stool samples were collected from patients attending the Donald Frazier, Elim, Tshilidzini, Siloam and Makhado hospitals.

### Isolation and identification of *Aeromonas* spp. from stool samples

In total, 309 stool samples were collected and investigated for *Aeromonas* spp. Microscopic examination was done with methylene blue stain for faecal leucocytes, red blood cells, and mucus. The specimens were cultured using the method as previously described (14). Briefly, freshly-collected stool specimens were plated onto MacConkey agar and xylose deoxycholate citrate agar (XDCA). Faeces were enrinched in peptone water (pH 8.6) overnight at 37 °C. Cultures were incubated at 37 °C for 18-24 hours, after which non-lactose-fermenting colonies on MacConkey agar and non-xylose-fermenting colonies on XDCA were screened for the production of oxidase. Oxidase-positive colonies were subcultured onto nutrient agar plates. All oxidase-positive colonies were further confirmed as *Aeromonas* using the API 20E system (Analytab Product). *Aeromonas* isolates were identified to species level using the following tests: esculin hydrolysis, Voges-Proskauer, lysine decarboxylase, growth in potassium cyanide broth, oxidation of gluconate to 2-keto-gluconate, gas production from glucose and glycerol, acid production from cellobiose, mannose, and lactose, and cell-free activity against rabbit erythrocytes (Tables 1 and 2).

**Table 1:** Scheme for identification of *Aeromonas* to species level

								Decarboxylsae			
Organism	Beta-haemolysis	Oxidase	Gas from glucose	Glycerol	Esculin	DNAse	Voges-Proskauer	Lysine	Ornithine	Arginine	Indole

*A. hydrophila*	+	+	+	+	+	+	+	+	-	+	+
*A. sobria*	+	+	+	+	-	+	+	+	-	+	+
*A. caviae*	-	+	-	-	+	+	-	-	-	+	+

**Table 2:** Scheme for identification of *Aeromonas* based on fermentation of acid from arabinose, cellobiose, lactose, and inositol

Organism	L-Arabinose	Cellobiose	Lactose	Inositol

*A. hydrophila*	+	-	-	-
*A. sobria*	-	-	-	-
*A. caviae*	+	+	+	-

### Determination of haemolytic activity

The previously-described methods were employed for haemolytic activity using sheep, human, horse, pig and chicken blood agar. A single colony of each isolate was streaked across blood agar plates using sterile inoculating wire loop. Plates were incubated at 37 °C for 18-24 hours. After incubation, the haemolytic activities were determined by observing haemolysis on blood agar.

### Determination of haemagglutination activity

The method of Atkinson and Trust was used for the detection of haemagglutinin (15). Erythrocytes were collected from human, horse, pig, and chicken into a bottle containing 5% EDTA and stored at 4 °C. Before use, they were washed three times in a 0.04-M phosphate-buffered saline (PBS) with ph 7.4, and a 3% suspension was prepared in PBS. Colonies of overnight cultures of *Aeromonas* on nutrient agar plates were incubated in Mueller-Hinton broth (Oxoid Ltd., London, England) for 18 hours at 37 °C. These cultures were centrifuged and washed twice in PBS. Haemagglutination tests were performed at room temperature by mixing 20 μL of erythrocyte suspension with 20 μL of bacterial suspension on a slide alongside a control suspension of erythrocytes and PBS and gently rocking by hand. Strains were considered haemagglutination-positive if agglutination occurred within five minutes and negative if agglutination was absent within this period.

### Testing of antibiotic susceptibility

Antibiotic susceptibility of the isolates was determined using the disc-agar diffusion technique (12). Several antibiotics were tested and were obtained from Oxoid; these included: ciprofloxacin (5 μg), erythromycin (30 μg), tetracycline (30 μg), meropenem (10 μg), imipenem (10 μg), chloramphenicol (10 μg), amoxacillin/clavulanic acid (30 μg), gentamicin (10 μg), amikacin (10 μg), cefoxitin (30 μg), nalidixic acid (30 μg), piperacillin/tazobactam (110 μg), doxycycline (30 μg), co-trimoxazole (25 μg), cefotaxime (30 μg), cephazolin (30 μg), cefuroxime (30 μg), cefepime (30 μg), and ceftrizone (30 μg). Briefly, five pure colonies of each bacterial strain were inoculated into 2 mL of sterile Mueller-Hinton broth in bijou bottles and incubated at 37 °C for six hours. The turbidity was adjusted to match a 0.5 McFarland turbidity standard. A sterile cotton-tipped swab was dipped into the standardized bacterial suspension, and the swab was rotated against the wall of the tube above the liquid level to remove excess inoculum. The inoculum was swabbed on the entire surface of a Mueller-Hinton agar plate. The automatic disc dispenser, adjusted to dispense six antibiotic discs, was applied on the surface of the agar, and the plates were incubated at 37 °C for 24 hours.

### Preparation of plants extracts and essential oils

The following medicinal plants were collected from the Venda region. *Bauhinia galpinii* (family Fabaceae), *Carissa edulis* (family Apocynaceae), *Ficus sycomorus* (family Moraceae), *Mormodica balsamina* (family Cucurbitaceae), *Syzygium cordatum* (family Myrtaceae). *Pterocarpus angolensis* (family Fabaceae), *Ximenia caffra* (family Olacaceae), and *Zornia milneana* (family Papilionaceae). A 50-g sample of each ground material was soaked in 500 mL of methanol, acetone, or hexane for at least 72 hours with frequent shakings. The samples were suction-filtered through Whatman No. 1 filter paper. The filtrate was evaporated to dryness under reduced pressure, collected in 10 mL of the solvent, placed in the tube and allowed to dry at room temperature. A stock solution of 0.2 g/mL in dimethyl sulphoxide (DMSO) was made for each extract. Essential oils were prepared by hydrodistillation for three hours using a Cleveland-type apparatus. All the extracts and essential oils were kept at 4 °C in the dark until further use as described (22).

### Antimicrobial assay of plant extracts

The disc-diffusion method was used as described by Nostro *et al.* (22). Briefly, Mueller-Hinton agar was supplemented with 0.01% tween 80 to enhance the solubilization of oils and extract. 100 μL of 18-hour old culture of each test organism was spread on the agar plate and left for 30 minutes to dry. Whatman paper was used for preparing discs of 6-mm diameter and sterilized by autoclaving. The blank sterile discs were deposited on top of the seeded Mueller-Hinton agar and 15 μL (3 mg) of each extract or essential oil was added on top of the disc. The plate was incubated at 37 °C for 24 hours. All tests were performed in triplicates using 10 μL of 50 mg/mL gentamycin as a positive control and 15 μL (6%) of DMSO as a negative control. Each test was repeated four times, and the antibacterial activity was expressed as the mean of inhibition diameters (mm) produced by the plant extracts (23).

### Determination of minimum inhibitoryconcentration

Serial dilutions of the extracts and essential oils were made in microtitre wells with Mueller-Hinton broth (0.01% tween 80) to cover the range of 0.08 mg/mL to 12 mg/mL for a volume of 100 μL with a final concentration of 2.5% DMSO (Merck, Germany). A McFarland no. 1 standard suspension of test bacteria was made in Mueller–Hinton broth, from which 100 μL of the final inoculum containing approximately 1.5×10^6^ colony-forming units (CFUs) was used for filling each well to a final volume of 200 μL. Inoculated plates were incubated at 37 °C for 24 hours. One hour before the end of incubation, 40 μL of a 0.2% solution of iodo-nitro tetrazolium (INT) (Merck, Germany) was added to the wells, and the plate was incubated for another hour. Inhibition of growth was detected when the solution in the well was clear after incubation with INT. The assay was repeated three times. The lowest concentration of each extract showing no visible growth was recorded as the minimum inhibitory concentration (MIC).

### Statistical analysis

The proportion difference was determined by the chi-square test. A p value of <0.05 was considered statistically significant.

## RESULTS

### Isolation rate of *Aeromonas*

**Fig. 1:**
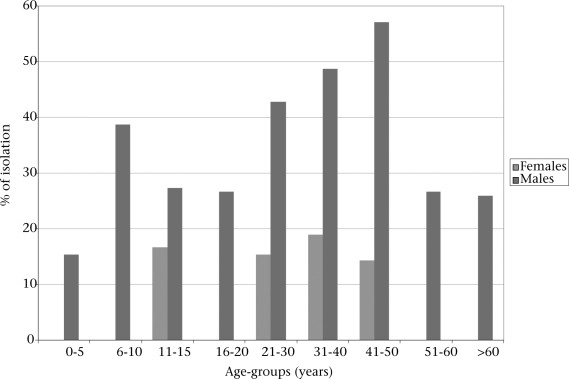
Age and sex distribution of *Aeromonas* species isolated in Vhembe district, South Africa

In total, 104 (33.66%) *Aeromonas* spp. were isolated from 309 samples screened. A breakdown of results showed that 89 (85.6%), 12 (11.5%), and three (2.9%) were *A. hydrophilia, A. sobria*, and *A. caviae* respectively. *A. hydrophilia* was the most frequentlyisolated species, and the difference was significant (p<0.05). The isolation rates of *Aeromonas* species were higher among male than among female patients (p<0.05). *A. hydrophila* was most commonly isolated from the age-group of 11-15 years and 31-40 years in females. No *Aeromonas* spp. was isolated in the age-group of 6-10 years and above 51 years in males (Fig. 1).

### Haemagglutinating and haemolytic activity

Haemagglutination activity of the *Aeromonas* strains tested showed 49% of activity against human red blood cells (HRBCs), 41% against sheep red blood cells (SRBCs), 26% against pig red blood cells (PRBCs), and 12% against chicken red blood cells (CRBCs) (Table 3). *A. hydrophila* demonstrated beta-haemolytic activity on all the red blood cells tested; 96%, 100%, 58%, and 85% to HRBCs, SRBCs, CRBCs, and PRBCs respectively; while *A. sobria* showed no haemolytic activity on HRBCs and SRBCs. All the *A. caviae* isolates showed beta-haemolysis on HRBCs and SRBCs (Table 4).

**Table 3:** Haemagglutination activities of *Aeromonas* spp. against human, sheep, pig, and chicken red blood cells

	Isolates showing haemagglutining activity	
Red blood cells	No.	%

HRBC	43	49
SRBC	36	41
PRBC	23	26
CRBC	11	12

CRBC=Chicken red blood cell; HRBC=Human red blood cell; PRBC=Pig red blood cell; SRBC=Sheep red blood cell

**Table 4:** Haemolytic activities of *Aeromonas* species on human, sheep, chicken, and pig red blood cells

Red blood cells	*A. hydrophila* (n=89)		*A. sobria* (n=12)		*A. caviae* (n=3)	
	Positive		Positive		Positive	
	No.	%	No.	%	No.	%

HRBC	85	96	0	-	3	100
SRBC	89	100	0	-	3	100
CRBC	52	58	1	8	0	-
PRBC	76	85	2	17	2	67

CRBC=Chicken red blood cell; HRBC=Human red blood cell; PRBC=Pig red blood cell; SRBC=Sheep red blood cell

### Antimicrobial susceptibility

**Fig. 2:**
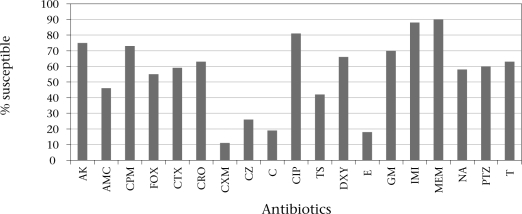
Antibiotic susceptibility of *Aeromonas* spp. isolated from diarrhoeic stools of patients attending different health centres in Vhembe district, Limpopo, South Africa

Results obtained showed a high incidence of resistance of the isolates to several antibiotics (Fig. 2). Resistance to cephalosporins was variable: 34% to cefotaxime, 79% to cefuroxime, and 26% to cefepime. Resistance to meropenem (7%), imipenem (11%), and ciprofloxacin (12%) was less observed.

AK=Amikacin; AMC=Amoxacillin/clavulanic acid; C=Chloramphenicol; CIP=Ciproflxacin; CPM=Cefepime; CRO=Ceftrizone; CTX=Ceftaxime; CXM=Cefuroxime; CZ=Cephazolin; DXY=Doxycycline; E=Erythromycin; FOX=Cefoxitin; GM=Gentamicin; IMI=Imipenem; MEM=Meropenem; NA=Nalidixic acid; PTZ=Piperacillin/tazobactum; T=Tetracycline; TS=Co-trimoxazole

### Medicinal plants

**Fig. 3:**
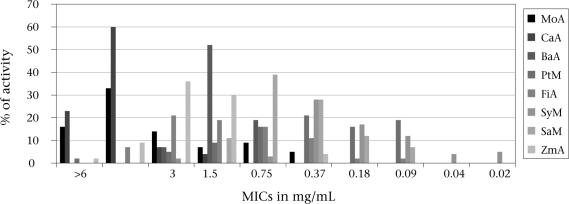
Inhibitory activity of acetone and methanol extracts of different plants against *Aeromonas* isolates

The results relating to the plant extracts are reported in Figure 3. Of eight extracts of eight different plants obtained, four exhibited activity against *Aeromonas* spp. isolates. Extracts of four plants showed little activity against the isolates with MIC of three mg/mL. The extracts of *P. angolensis*, *S. cordatum*, and *Z. milneana* using acetone and hexane as the extractant showed a significant inhibitory effect against all the strains of *Aeromonas* spp. tested.

CaA=*Carissa edulis* using acetone as an extractant; BaA=*Bauhinia galpinii* using acetone as an extractant; FiA=*Ficus sycomorus* using acetone as an extractant; MICs=Minimum inhibitory concentrations; MoA=*Mormodica balsamina* using acetone as an extractant; PtM=*Pterocarpus angolensis* using methanol as an extractant; SaM=*Ximenia caffra* using methanol as an extractant; SyM=*Syzygium cordatum* using methanol as an extractant; ZmA=*Zornia milneana* using acetone as an extractant. **Key:** *Bauhinia galpinii* (family Fabaceae), *Carissa edulis* (family Apocynaceae), *Ficus sycomorus* (family Moraceae), *Mormodica Balsamina* (family Cucurbitaceae), *Syzygium cordatum* (family Myrtaceae). *Pterocarpus angolensis* (family Fabaceae), and *Zornia milneana* (family Papilionaceae)

## DISCUSSION

It has been reported that there is a great variability in the isolation and distribution of *Aeromonas* species from clinical samples, particularly from stool specimens (3). According to Maluping *et al.*, *Aeromonas* spp. were responsible for more dysentery than *Shigella* species (24). Studies elsewhere revealed that 12.3% of 210 patients screened harboured *Aeromonas* spp. in their faeces (18). In the present study, 33% of the diarrheic stool samples examined were positive for *Aeromonas* spp. Although results of some studies showed that *Aeromonas* spp. were more prevalent in children aged less than five years, the present study recorded a higher occurrence in adults aged 21-50 years. The higher prevalence (33%) underlines the need to include *Aeromonas* spp. in the list of routinely-analyzed enteropathogens in all diarrhoeal stool samples as previously suggested (14). Our results showing the incrimination of *A. hydrophila, A. sobria*, and *A. caviae* in diarrhoeic cases are consistent with results of previous reports (1,2,7,17) and justify further studies, such as determination of pathogenic indices and antimicrobial activities.

In the present study, *A. caviae* and *A. hydrophilia* showed high haemolytic activity on human and sheep red blood cells. Several authors have shown that production of haemolysin is mainly associated with strains belonging to the phenospecies—*A. hydrophila* and *A. sobria* (13,14,17). However, in contrast to these reports, *A. sobria* isolates in this study showed no haemolytic effect on either sheep or human red cells. This indicates a variation in haemolytic activities of *Aeromonas* species and could be linked to the sources of the *Aeromonas* isolates. The present study investigated *Aeromonas* spp. in clinical samples, while previous authors studied environmental isolates, such as from water and food. These variations may indicate the need for detailed studies on the pathogenicity and relationships between environmental and clinical isolates of *Aeromonas* spp.

Haemolytic activity is an index of pathogenicity, and the relationship between the production of haemolysin and the enterotoxigenicity in *Aeromonas* is well-documented (14,15,17). The reported haemolytic activities of the isolates in the present study point to the potential pathogenic significance, although haemolytic activities varied with species.

Erythrocytes from small laboratory animals are more sensitive than human, horse, or sheep erythrocytes in *Aeromonas* haemoylsin and haemagglutinins assay (16). However, if the haemoylsin assay is to be used routinely in clinical laboratories to detect enteropathogenic aeromonads, small animals are not practical sources of erythrocytes because of difficulty in obtaining blood from these animals. The result of this study has demonstrated that human, horse, or sheep red blood cells may be adequate for haemolysin and haemagglutination assay of *Aeromonas*. This finding is in harmony with reports of other investigators (13,17). Although pig and chicken erythrocytes had not been previously reported to detect haemolytic and haemagglutinin activities of *Aeromonas* spp., they offer promise in the detection of haemagglutinating and haemolytic activities of *Aeromonas* species, as exemplified by results of this study.

Other major pathogenic features, such as cytotoxic enterotoxin (Act) and Type111 secretion system, had been described for *Aeromonas* (25) but were not investigated in the present study. A major observation of the present study was the demonstration of multiple antibiotic resistance among *Aeromonas* isolates. This poses a major public-health concern and calls for concerted efforts to unravel alternative sources of treatment. Medicinal plants offer such promise. Results have revealed the strong in-vitro activity of *P. angolensis, S. cordatum*, and *Z. milneana* against *Aeromonas* isolates. Medicinal plants are useful sources of drugs (26,27), and the majority of individuals in developing countries rely on their uses. Medicinal plants offer cheaper and more natural remedies to infections. Structural elucidation of the active components of the plants may be a prelude to the discovery of candidate drug templates for eventual drug designs and development.

It is concluded that diarrhoeagenic *Aeromonas* species were commonly encountered in the region studied and could be the cause of diarrhoea in both children and adults. Although species of *Aeromonas* studied showed haemagglutinating and haemolytic activities, these may not be conclusive of their pathogenicity. Other major pathogenic factors, such as heat-labile cytotoxin, heat-stable cytotoxin that have enterotoxic activities, will be the subject of another investigation.

In the empiric management of *Aeromonas*-related infections, ciprofloxacin, meropenem, and imipenem may be effective as demonstrated by results on antibiograms. Further studies are needed to fully confirm the strong antimicrobial activity observed for *P. angolensis, S. cordatum*, and *Z. milneana* against *Aeromonas* isolates.
